# Subthreshold depression prevalence and influencing factors among nursing students in vocational schools: A cross-sectional study

**DOI:** 10.1371/journal.pone.0346701

**Published:** 2026-04-20

**Authors:** Ruoying Cheng, Wei Zhu, Shuyu Jiang, Zijuan Yang

**Affiliations:** 1 Nursing Department, Wuxi Higher Health Vocational Technology School, Jiangsu Union Technical Institute, Wuxi, Jiangsu Province, PRC; 2 Psychological Department, The Affiliated Wuxi Mental Health Center of Jiangnan University, Wuxi, Jiangsu Province, PRC; 3 Psychological Counselling Center, Wuxi Higher Health Vocational Technology School, Jiangsu Union Technical Institute, Wuxi, Jiangsu Province, PRC; Universidad Técnica de Manabí: Universidad Tecnica de Manabi, ECUADOR

## Abstract

**Background:**

Subthreshold depression (SD) is defined as a subclinical symptomatic state characterized by depressive symptoms that do not meet diagnostic criteria yet still impair quality of life and functioning. The prevalence of this condition is notably high among adolescents. Vocational nursing students, as a vital component of healthcare provision, face a range of pressures. Consequently, the incidence of subthreshold depression among this cohort and its contributing factors warrant careful consideration.

**Objectives:**

We aimed to investigate the prevalence of subthreshold depression among vocational nursing students in eastern China and to explore the related factors.

**Methods:**

Purposive sampling was employed to select nursing students from nine vocational colleges in eastern China. Data on respondents’ general characteristics, family type, cognitive attitudes, social support, resilience, personality traits, and depression status were gathered to ascertain the correlation between each variable and the associated factors of subthreshold depression. Multivariable binary logistic regression was utilised to formulate a regression model that elucidated the factors associated with subthreshold depression among vocational nursing students.

**Results:**

The prevalence rate for subthreshold depression among vocational nursing students was found to be 54.3%. SD students showed relatively unhealthy cognitive attitudes and heightened emotional sensitivity and instability, as well as lower levels of family functioning, social support, and psychological resilience. Logistic regression analysis revealed that among respondents, female gender, DAS and CBF-PI-B-Neuroticism trait were independently and positively associated with SD after adjustment for confounders. In contrast, social support and resilience were independently and negatively associated with SD (*P* < 0.05).

**Conclusions:**

Students at vocational nursing schools exhibit a relatively high prevalence of subthreshold depression, with multiple contributing factors. Various stakeholders, including the state, communities, hospitals, academic institutions, and families, should develop and share resources to develop relevant programmes to improve the mental health of vocational nursing students in China.

## 1. Introduction

Subthreshold depression (SD), also termed subclinical or sub-syndromal depression, denotes the presence of depressive symptoms that do not meet the diagnostic criteria for major depressive disorder (MDD) [1]. The condition is frequently accompanied by persistent fatigue, impaired memory, insomnia, and difficulty concentrating, and it may even involve suicidal tendencies. It is frequently regarded as a precursor to major depressive disorder [2,3].

In recent decades, subthreshold depression has been the focus of mounting attention. Currently, there is no consensus on the definition of subthreshold depression in terms of the number, frequency, and duration of symptoms. According to epidemiological studies, the morbidity of subthreshold depression in the population is 14–30% [4,5]. Approximately 29.2% of adolescents are affected by SD, with the prevalence of this condition increasing steadily between the ages of 12 and 20 [6,7]. Furthermore, between 10% and 20% of patients with subthreshold depression will convert to MDD [8]. Although subthreshold depression is characterised by a lower severity rating than that of MDD, it nevertheless exerts a significant impact on quality of life, resulting in increased functional disability and mortality. Moreover, it constitutes a considerable physical, mental, and economic burden.

The mental health status of medical staff influences their professional competence, as well as service quality, career development, and even the maintenance of a harmonious nurse-patient and doctor-patient relationship. For nursing students, there mental health not only affects their daily life and academic performance but also has important implications for future clinical practice. As prospective healthcare providers, maintaining a sound psychological state is essential to safeguarding the physical and mental health of patients. Previous studies have shown that the prevalence of subthreshold depression among Chinese college students is close to 40% [9]. Due to the heavy academic burden and the high pressure of their future professional environment, the detection rate of subthreshold depression in medical students is as high as 69%, which is much higher than that in the general population [10].

Eastern China is one of the most economically advanced regions in the country, with one of the top levels of educational development. Within this context, this region delivers a substantial amount of higher professional education in nursing and cultivates high-calibre talent. However, vocational education, as a relatively distinctive category of talent development, is characterised by a relatively lower academic positioning, and its graduates often face suboptimal employment outcomes-this issue is particularly prominent in the economically prosperous Yangtze River Delta region of China. Furthermore, vocational school students are in the adolescent stage, a pivotal period marked by encounters with numerous new challenges, role transitions, and developmental tasks [11]. The co-occurrence of critical life events and a relatively insufficient level of social or material resources renders this stage a high-risk window for the onset of mental disorders [12]. In recent years, the mental health status of this student cohort has raised growing concerns, as poor mental health causes adverse impacts on the development and subsequent harnessing of health-related talents. However, no relevant research has yet been documented in the literature.

Against this backdrop, it is imperative to conduct a comprehensive screening for subthreshold depression among nursing students in vocational schools in eastern China. Such an initiative will allow us to identify effective mechanisms of action and develop targeted intervention strategies, thereby enabling early intervention in the target population. Ultimately, our aim is to enhance the mental health of future medical professionals and further improve the overall quality of the workforce in this field.

## 2. Materials and methods

### 2.1. Design

A cross-sectional study was conducted to investigate the prevalence of subthreshold depressive symptoms and their associated factors among nursing students enrolled in medical vocational schools in Eastern China.

This study was reviewed and approved by the Medical Research Ethics Committee of Wuxi Higher Health Vocational Technology School (Approval Number: WXWX [20210908]). All research procedures were implemented in strict accordance with the ethical principles outlined in the Declaration of Helsinki and the regulatory guidelines of relevant governing authorities. Prior to study participation, all subjects were informed of the study details and voluntarily provided written informed consent. Throughout the processes of data collection, statistical analysis, and the reporting of results, strict anonymisation was applied to protect participants’ privacy. Additionally, targeted measures were adopted to ensure that no physical, psychological, or social harm inflicted on any research participant.

### 2.2. Participants

#### 2.2.1. Sample source.

The subjects of this study were nursing students in vocational schools in Eastern China. From March to May 2025, purposive sampling was used to select nursing students from 9 medical vocational schools, and we distributed the “Wenjuanxing” (an online survey platform) link to complete the questionnaire. A total of 1,588 questionnaires were collected, with 1,467 valid responses.

#### 2.2.2. Inclusion criteria.

The inclusion criteria included the following:

- Chinese nationality;
- Nursing students currently enrolled in vocational schools;
- Informed consent and voluntary participation in this study;
- The respondent completed the questionnaire by themselves or with assistance from the investigator.

#### 2.2.3. Exclusion criteria.

A total of 1467 valid response questionnaires were collected, and invalid questionnaires were excluded for the following reasons:

- The respondent had been diagnosed with severe mental or physical illness or had limited mobility;
- Questionnaire with missing items;
- Questionnaires showed incorrect answers to ≥2 of the 3 ’trap questions’;
- Questionnaires were completed in less than 180s.

### 2.3. Research instruments

The research instruments used in this study were categorised into 3 components. The first investigated the general characteristics of the respondents. The second investigated the depression status of the respondents using the Center for Epidemiological Survey Depression Scale (CES-D). The third comprised a series of other standard scales, including the Family Adaptability and Cohesion Scale-Chinese Version (FACES-II-CV), Dysfunctional Attitude Scale (DAS), Chinese Adolescent Social Support Scale (CASS), 10-item Connor–Davidson Resilience Scale (CD-RISC-10), and Chinese Big Five Personality Inventory brief version (CBF-PI-B). The instruments were used to investigate respondents’ family situation, the presence and intensity of dysfunctional attitudes, and their social support status, mental resilience, and personality traits.

#### 2.3.1. Center for Epidemiological Survey Depression Scale (CES-D).

Depressive symptoms were assessed using the Center for Epidemiological Survey Depression Scale (CES-D) [13], which is by far the most frequently used instrument [14]. The CES-D is a 20-item self-reported questionnaire that evaluates depressive symptoms experienced over the past week (i.e., the recall period is restricted to the preceding 7 days). Comparative analyses with the Hamilton Depression Rating Scale (HAMD) have demonstrated that the CES-D exhibits good validity; thus, it is suitable for application in epidemiological investigations. In this study, the Chinese version of the scale translated by Zhang [15] was adopted. Its reliability and validity had been examined among Chinese adolescents by Chen [16], with a Cronbach’s *α* coefficient of 0.88 and an average inter-item correlation of 0.26, indicating satisfactory reliability. In large-scale mental health surveys, the CES-D is typically employed as an initial screening instrument: following initial screening, individuals with positive results undergo further diagnostic assessments to confirm potential depressive conditions. According to general research consensus, the CES-D scoring criteria are defined as follows: a score of ≤15 indicates the absence of depressive symptoms; a score ranging from 16 to 19 suggests the possible presence of depressive symptoms; and a score of ≥20 denotes the definite presence of depressive symptoms.

In SD studies, two differing approaches are taken to the cut-off value for SD: the most commonly used definition for SD using CES-D is CES-D ≥ 16 [14,17], but sometimes, a CES-D score≥20 serves as the initial screening threshold [18]. There is no definitive evidence indicating that one cut-off value is superior to the other. Using the commonly applied cutoff for SD in prior research, participants with a CES‑D score≥16 were classified as having SD, while those with a score≤15 were classified as the control group in this study.

#### 2.3.2. Family Adaptability and Cohesion Scale—Chinese Version (FACES II-CV).

The Family Adaptability and Cohesion Scale—Chinese Version (FACES II-CV) consists of 30 items and is divided into 2 dimensions: family cohesion and family adaptability [19,20]. According to the ‘arch model’ [21], the subject families are divided into 16 family types. Those who are in the middle of the 4 types (i.e., Flexible-Connected, Structured-Connected, Structured-Separated, Flexible-Separated) on the two subscales are balanced, showing a moderate emotional distance between family members, both intimate and independent; that is, they have well-adapted, healthy families. Those who are at the extremes of the 4 types (i.e., Chaotic-Disengaged, Rigid-Disengaged, Chaotic-Enmeshed, Rigid-Enmeshed) on the two subscales show extreme levels of family cohesion and adaptability, that is, the lowest level or the highest level, and these families and their members often show diametrically opposite characteristics in intimacy and adaptability. The other 8 types (i.e., Flexible-Disengaged, Structured-Disengaged, Chaotic-Separated, Rigid-Separated, Chaotic-Connected, Rigid-Connected, Flexible-Enmeshed, Structured-Enmeshed) are intermediate on one subscale and extreme on the other and are classified as being of an intermediate type.

FACES Ⅱ is widely used in Western countries. Fei [22] translated the scale into Chinese, revised it three times, and assessed its reliability and validity within Chinese families. The results showed that the scale demonstrated good content validity and internal consistency, and Cronbach’s α coefficients for the two dimensions were 0.67 and 0.72, respectively.

#### 2.3.3. Dysfunctional Attitude Scale form A (DAS-A).

The Dysfunctional Attitude Scale form A (DAS-A) is a self-report scale designed to measure the presence and intensity of dysfunctional attitudes [23]. The DAS-A consists of 40 items, and each item consists of a statement and a 7-point Likert scale (7 = fully agree; 1 = fully disagree). The total score is the sum of the 40 items, with a range of 40–280. The higher the score, the more dysfunctional attitudes an individual possesses [24]. The 40 items can be divided into eight dimensions: Vulnerability, Perfectionism, Approval and Disapproval, Compulsivity, Dependence, Autonomy Attitudes, Need for Approval, and Cognitive Philosophy. The higher the total score, the more severe the cognitive distortion. Cai [25] brought together clinical psychologists and bilingual linguists from both China and Canada to conduct a series of iterative translations and back-translations of all items, ultimately creating the Chinese version of the Dysfunctional Attitude Scale. This scale underwent pilot testing and validation with Chinese adolescents, yielding a total scale reliability coefficient of 0.88 and a test-retest reliability coefficient of 0.84.

#### 2.3.4. Chinese Adolescent Social Support Scale (CASS).

The Chinese Adolescent Social Support Scale (CASS) was developed by Ye [26] in 2008. It consists of 17 items, including three dimensions, subjective support, objective support, and support utilisation, and is used to assess the extent to which individuals receive and utilise social resources. This instrument uses a 5-point Likert scale (1 = not applicable, 2 = somewhat not applicable, 3 = indeterminate, 4 = somewhat applicable, and 5 = applicable). The higher the score, the more social support the individual has. According to the total score on the scale, the level of support received is divided into 3 categories: low (total score<50), medium (50–77), and high (>77). The internal consistency of the whole scale is 0.907, and the reliability of each subscale is 0.846, 0.806, and 0.838, respectively. The KMO is 0.915, indicating good structural validity. In this study, Cronbach’s α coefficient for this scale was 0.77.

#### 2.3.5. 10-item Connor–Davidson Resilience Scale (CD-RISC-10).

The Connor-Davidson Resilience Scale (CD-RISC) was developed by Connor and Davidson [27]. Campbell-Sills [28] simplified the scale, resulting in the 10-item CD-RISC-10. Subsequently, Yu [29] translated and culturally adapted this 10-item version into Chinese, establishing the Chinese version of the CD-RISC-10, and validated it in Chinese populations. CD-RISC-10 (Chinese version) scored using a 5-point Likert scale (0 = Never, 1 = Rarely, 2 = Sometimes, 3 = Often, 4 = Always). The scale initially included 5 factors: tenacity, tolerance of negative affect, positive acceptance of change, control, and spiritual beliefs. It includes the 3 dimensions of tenacity, strength, and optimism and has good psychometric properties. This study used a 10-item short version of the Resilience Scale, extracting items 1, 4, 6, 7, 8, 11, 14, 16, 17, and 19 from the original 25 items, with a total score ranging from 0 to 40. Higher scores reflect higher levels of resilience. Cronbach’s α coefficient for this study was 0.942.

#### 2.3.6. Chinese Big Five Personality Inventory—brief version (CBF-PI-B).

Taking the Big Five model as the theoretical framework and building upon the Big Five Inventory (BFI) developed by John [30], Wang [31,32] compiled a Chinese version of the Big Five Personality Inventory. Through statistical methods, they further selected appropriate items to form a short version, namely the Chinese Big Five Personality Inventory-Brief Version (CBF-PI-B). This inventory consists of 40 items and including five dimensions: CBF-PI-B-Openness, CBF-PI-B-Conscientiousness, CBF-PI-B-Extraversion, CBF-PI-B-Agreeableness, and CBF-PI-B-Neuroticism. Each dimension is measured using 8 items and a 6-point Likert scale (1 = very inconsistent, 2 = relatively inconsistent, 3 = slightly inconsistent, 4 = slightly consistent, 5 = relatively consistent, 6 = very consistent). The correlation between each factor in the brief version (CBF-PI-B) and the corresponding factor in the full version (CBF-PI) is above 0.85. Cronbach’s α coefficient for the five dimensions of the brief version is above 0.75 [33].

### 2.4. Data analysis

Data entry and analysis were performed using SPSS™ for Windows (version 26.0). Categorical variables were expressed as frequencies (percentages) and compared using the *Chi-squared* test. The Kolmogorov-Smirnov (K-S) test was used to assess the normality of continuous variables. Normally distributed data were presented as mean±standard deviation(*M* ± *SD*) and compared using independent-samples *t*-test. Non-normally distributed data were presented as median (IQR) and analysed using the Mann-Whitney U test. Based on theoretical rationale, previous evidence regarding the influences of individual, family, and social factors on depression [34–38], we conducted a multivariable analysis of the factors associated with subthreshold depression among the respondents using multivariable binary logistic regression (forced entry method). The results are presented as odds ratios (*OR*) with 95% confidence intervals (95% *CI*). To ensure reliable results, we evaluated the internal consistency reliability of the scales used and assessed the potential influence of common method bias using the Harman one-way method. Unless otherwise stated, the level of statistical tests was *α* = 0.05, and all tests were two-sided.

## 3. Results

### 3.1. Common method variance test

To mitigate potential common method bias, procedural remedies, including anonymous participation and standardized instructions, were implemented during data collection. Subsequently, a Harman’s single-factor test was conducted on all items using unrotated exploratory factor analysis. The first factor explained 23.53% of the total variance, which is below the conventional threshold of 40%, indicating the absence of a dominant common factor. While this suggests that common method bias was unlikely to be a serious concern in the present study, it is important to acknowledge that Harman’s test provides only a preliminary assessment. Future studies are encouraged to employ more robust methodological approaches to further validate the findings.

### 3.2. Normality test

In this study, the Kolmogorov-Smirnov test (*P* < 0.05) revealed that continuous variables exhibited a non-normal distribution. However, visual inspection using quantile-quantile (Q-Q) plots demonstrated that data points were predominantly within a tolerable range around the diagonal line. This finding suggests that, although the data deviated from normality, there was an absence of severe skewness or outliers. In order to enhance the robustness of the findings, results from both parametric (independent-samples *t*-test) and non-parametric (Mann-Whitney U test) tests were reported.

### 3.3. General characteristics and subthreshold depressive status of respondents

A total of 1,589 questionnaires were collected in this study. After screening, 1,467 valid responses remained (92.32%). The effective respondents fell within the 15–20 age range (17.5 ± 3.7). The majority of respondents were female, from vocational schools (90.7%). Half of the respondents lived in urban areas. The majority were the only children in their family (53.9%). Respondents’ parents were mostly working or self-employed, mainly had a high school education, and were generally married (85.6%). Regarding the FACES-II-CV scale, the majority of this sample were ‘mid-range’ (42.1%).

Among the 1,467 valid questionnaires, 797 students met the criteria for subthreshold depression, yielding a point prevalence rate of 54.3%. Compared to their non-depressed counterparts, students with subthreshold depression exhibited significant demographic differences: female students demonstrated a significantly higher prevalence rate than male students (*χ*² = 8.759, *P* = 0.003); prevalence was higher among those whose mothers had attained a lower level of education (*χ*² = 10.665, *P* = 0.005); parental marital status significantly influenced prevalence (*χ*² = 10.574, *P* = 0.005); and significant differences were observed based on family type, with the highest prevalence found in mid-range families (*χ*² = 58.284, *P* < 0.001) (Table 1.).

**Table 1:** Comparison of subthreshold depression prevalence among 1,467 vocational college nursing students across different characteristics.

Variables	N(%)	Non-SD[n(%)]	SD[n(%)]	*χ*²	*P*
**Total**	1467 (100)	670 (45.7)	797 (54.3)		
**Gender**				8.759	0.003**
Male	137 (9.3)	79 (11.8)	58 (7.3)		
Female	1330 (90.7)	591 (88.2)	739 (92.7)		
**Grade**				7.585	0.108
Grade 1	453 (30.9)	190 (28.4)	263 (33.0)		
Grade 2	433 (29.5)	210 (30.0)	232 (29.1)		
Grade 3	329 (22.4)	165 (24.6)	164 (20.6)		
Grade 4	182 (12.4)	77 (11.5)	105 (13.2)		
Grade 5	70 (4.8)	37 (5.5)	33 (4.1)		
**Residence**				3.651	0.056
Rural	734 (50.0)	317 (47.3)	417 (52.3)		
Urban	733 (50.0)	353 (52.7)	380 (47.7)		
**Father’s occupation**				3.043	0.385
Public sector workers	124 (8.5)	53 (7.9)	71 (8.9)		
Blue-collar worker	687 (46.8)	318 (47.5)	369 (46.3)		
Self-employed	617 (42.1)	286 (42.7)	331 (41.5)		
Unemployed	39 (2.7)	13 (1.9)	26 (3.3)		
**Mother’s occupation**				2.312	0.510
Public sector workers	98 (6.7)	45 (6.7)	53 (6.6)		
Blue-collar worker	720 (49.1)	342 (51.0)	378 (47.4)		
Self-employed	529 (36.1)	233 (34.8)	296 (37.1)		
Unemployed	120 (8.2)	50 (7.5)	70 (8.8)		
**Father’s level of education**				4.638	0.098
Lower secondary or below	552 (37.6)	234 (34.9)	318 (39.9)		
Upper secondary	723 (49.3)	350 (52.2)	373 (46.8)		
Bachelor’s degree or higher	192 (13.1)	86 (12.8)	106 (13.3)		
**Mother’s level of education**				10.665	0.005**
Lower secondary or below	628 (42.8)	256 (38.2)	372 (46.7)		
Upper secondary	690 (47.0)	341 (50.9)	359 (43.8)		
Bachelor’s degree or higher	149 (10.2)	73 (10.9)	76 (9.5)		
**Only child (yes/no)**				2.228	0.136
Yes	790 (53.9)	375 (56.0)	415 (52.1)		
No	677 (46.1)	295 (44.0)	382 (47.9)		
**Marital status of parents**				10.574	0.005**
Intact family	1256 (85.6)	589 (87.9)	667 (83.7)		
Divorced/separated	180 (12.3)	75 (11.2)	105 (13.2)		
Widowed	31 (2.1)	6 (0.9)	25 (3.1)		
**Family type**				58.284	<0.001***
Mid-range	617 (42.1)	352 (39.1)	355 (44.5)		
Flexible–Disengaged	0 (0)	0 (0)	0 (0)		
Structured–Disengaged	6 (0.4)	4 (0.6)	2 (0.3)		
Chaotic–Separated	0 (0)	0 (0)	0 (0)		
Rigid–Separated	158 (10.8)	26 (3.9)	132 (16.6)		
Chaotic–Connected	3 (0.2)	2 (0.3)	1 (0.1)		
Rigid–Connected	79 (5.4)	30 (4.5)	49 (6.1)		
Flexible–Enmeshed	284 (19.4)	162 (24.2)	122 (15.3)		
Structured–Enmeshed	87 (5.9)	38 (5.7)	49 (6.1)		
Balanced	351 (23.9)	116 (17.3)	235 (29.5)		
Flexible–Connected	148 (10.1)	47 (7.0)	101 (12.7)		
Structured–Connected	148 (10.1)	50 (7.5)	98 (12.3)		
Structured–Separated	47 (3.2)	14 (2.1)	33 (4.1)		
Flexible–Separated	8 (0.5)	5 (0.7)	3 (0.4)		
Extreme	499 (34.0)	292 (43.6)	207 (26.0)		
Chaotic–Disengaged	0 (0)	0 (0)	0 (0)		
Rigid–Disengaged	126 (8.6)	17 (2.5)	109 (13.7)		
Chaotic–Enmeshed	361 (24.6)	272 (40.6)	89 (11.2)		
Rigid–Enmeshed	12 (0.8)	3 (0.4)	9 (1.1)		

*P < 0.05; ** *P* < 0.01; *** *P* < 0.001.

### 3.4. Analysis of factors contributing to subthreshold depression among vocational nursing students

As shown in Table 2, compared to the control group with a normal level of mental health, SD group exhibited significantly higher scores on the total DAS and all its subscales. Conversely, the SD group demonstrated significantly lower levels of family cohesion and adaptability, social support (including all its subdimensions), and psychological resilience. Regarding personality traits, the SD group scored lower for all factors with the exception of Neuroticism, for which they scored significantly higher. Similar results were obtained using both independent-samples *t*-test and Mann-Whitney U test, and all these differences were statistically significant (*P* < 0.001).

**Table 2:** Scale score grading and t-test results of the respondents.

Variables		*t-test* (*M* ± *SD*)			Mann-Whitney U [*M*(IQR)]		
		Non-SD	SD	*t*	Non-SD	SD	*Z*
DAS	Total Score	120.30 ± 24.836	147.48 ± 26.869	−19.978^***^	120.00 (33)	147.00(34)	−17.944^***^
	Vulnerability	17.32 ± 3.064	19.54 ± 3.049	−13.913^***^	17.00(5)	20.00(3)	−13.385^***^
	Attraction/Repulsion	15.28 ± 4.591	19.35 ± 4.926	−16.284^***^	15.00(7)	19.00(6)	−14.910^***^
	Perfectionism	14.73 ± 4.899	17.44 ± 3.679	−17.417^***^	14.00(7)	19.00(6)	−16.050^***^
	Compulsivity	15.76 ± 3.454	18.10 ± 3.518	−12.763^***^	16.00(5)	18.00(4)	−12.247^***^
	Need for Approval	12.77 ± 4.312	16.56 ± 5.103	−15.187^***^	13.00(6)	16.00(7)	−13.908^***^
	Dependence	14.59 ± 4.342	18.24 ± 4.202	−16.322^***^	15.00(6)	18.00(5)	−15.148^***^
	Autonomous Attitudes	16.25 ± 5.234	20.31 ± 5.409	−14.560^***^	16.00(7)	20.00(7)	−13.500^***^
	Cognitive Philosophy	13.60 ± 4.358	16.09 ± 4.043	−11.270^***^	13.00(5)	16.00(6)	−11.632^***^
Family Cohesion		75.97 ± 9.587	66.78 ± 10.883	17.180^***^	77.00(13)	68.00(14)	−16.270^***^
Family Adaptability		54.75 ± 8.840	46.68 ± 9.876	16.502^***^	55.00(12)	48.00(12)	−15.370^***^
Social Support		75.88 ± 10.754	66.22 ± 12.773	15.728^***^	80.00(16)	68.00(18)	−15.516^***^
Perceived Social Support		21.95 ± 3.607	18.89 ± 4.291	14.845^***^	30.00(4)	25.00(7)	−14.364^***^
Objective Social Support		27.76 ± 3.648	24.66 ± 4.837	13.974^***^	23.00(5)	19.00(6)	−14.661^***^
Utilisation of Support		26.17 ± 4.463	22.67 ± 5.129	13.975^***^	28.00(6)	24.00(7)	−13.987^***^
Resilience		28.80 ± 7.023	22.53 ± 6.360	17.937^***^	29.00(9)	22.00(8)	−17.062^***^
CBF-PI-B-Openness		36.07 ± 7.471	33.64 ± 6.753	6.482^***^	36.00(8)	33.00(9)	−6.864^***^
CBF-PI-B-Conscientiousness		35.54 ± 6.830	31.54 ± 5.848	11.921^***^	36.00(9)	31.00(7)	−11.943^***^
CBF-PI-B-Extraversion		32.83 ± 7.011	29.65 ± 6.172	9.131^***^	33.00(9)	30.00(8)	−9.168^***^
CBF-PI-B-Agreeableness		36.27 ± 6.013	33.22 ± 5.611	9.972^***^	36.00(8)	33.00(7)	−9.936^***^
CBF-PI-B-Neuroticism		19.88 ± 6.730	29.46 ± 6.638	−27.342^***^	19.00(10)	30.00(9)	−22.764^***^

* *P* < 0.05; ** *P* < 0.01; *** *P* < 0.001.

* Scores for all variables were obtained from general characteristics, CES-D, FACES-II-CV, DAS, CASS, CD-RISC-10 and CBF-PI-B.

**Fig 1:**
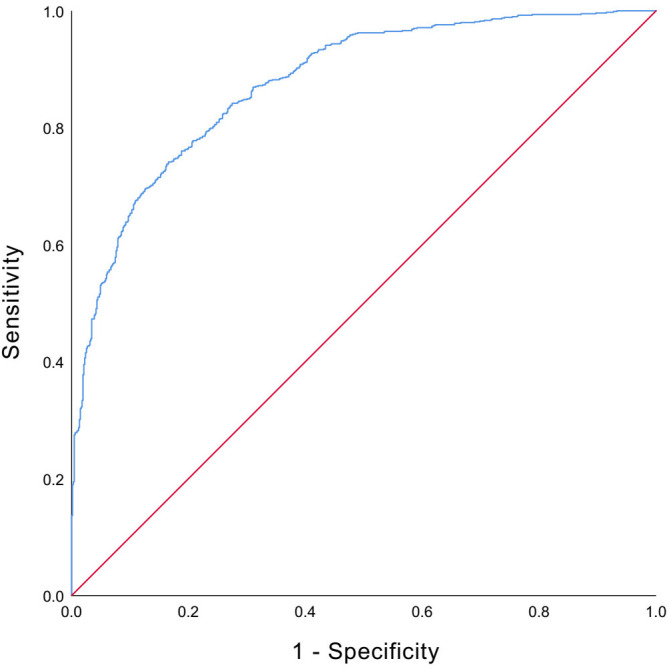
Receiver operating characteristic (ROC) curve of the multivariable binary logistic regression model. The model achieved an AUC of 0.875 (95% *CI*: 0.858-0.893, *P* < 0.001).

Multivariable binary logistic regression analysis was performed to identify independent influencing factors. The overall model was statistically significant (Omnibus test: *χ*² = 736.295, *df* = 33, *P* < 0.001). The −2 log-likelihood was 1286.391, and the Nagelkerke *R*² was 0.527. The Hosmer–Lemeshow test indicated excellent model calibration (*χ*² = 7.461, *df* = 8, *P* = 0.488). The model demonstrated good discrimination, with an area under the ROC curve (AUC) of 0.875 (95% *CI*: 0.858–0.893, *P* < 0.001) (Fig 1).

Taking subthreshold depression as the dependent variable, a binary logistic regression analysis was performed using the forced-entry method. Independent variables were selected based on theoretical rationale, prior research, and clinical relevance, including sociodemographic characteristics and total scores of each scale, with no variables excluded according to univariate test results. In order to circumvent multicollinearity, only the total score of each scale was included in the regression analysis, while subdomain scores were not entered simultaneously. These variables included respondent’s gender, grade, residence, parental occupation, parental educational level, parental marital status, only-child or not, family type, DAS score, family cohesion, family adaptability, social support, resilience, and the five factors in the CBF-PI-B. As shown in Table 3, the results indicated that gender, DAS, Social Support, Resilience and the CBF-PI-B-Neuroticism were retained in the final regression model. In particular, female gender (*OR*=2.862, 95% *CI* = 1.739–4.711, *P* < 0.001), DAS (*OR*=1.012, 95% *CI* = 1.005–1.019, *P* < 0.01), and CBF-PI-Neuroticism trait (*OR*=1.164, 95% *CI* = 1.134–1.195, *P* < 0.001) were independently and positively associated with SD after adjustment for confounders. In contrast, social support (*OR*=0.977, 95% *CI* = 0.963–0.991, *P* < 0.01) and resilience (*OR*=0.970, 95% *CI* = 0.944–0.998, *P* < 0.05) were independently and negatively associated with SD. All 95% confidence intervals excluded 1, supporting the statistical reliability of these independent associations. These findings extended univariate results and indicated that the above variables were robust and independent correlates of subthreshold depression.

**Table 3:** Multivariable binary logistic regression results for factors associated with subthreshold depression among vocational nursing students in eastern China.

Variable	*B*	*SE (B)*	Wald *χ*²	*P*	*OR*	95% *CI*	
						Lower	Upper
Gender (Ref: Male)							
Female	1.052	0.254	17.113	<0.001^***^	2.862	1.739	4.711
Grade (Ref: Grade 1)							
Grade 2	0.014	0.183	0.005	0.941	1.014	0.708	1.452
Grade 3	−0.166	0.194	0.731	0.393	0.847	0.579	1.239
Grade 4	0.270	0.242	1.241	0.265	1.309	0.815	2.104
Grade 5	0.328	0.351	0.873	0.350	1.388	0.698	2.763
Residence (Ref: Rural)							
Urban	−0.100	0.154	0.426	0.514	0.905	0.669	1.223
Father’s occupation (Ref: Public sector workers)							
Blue-collar worker	−0.252	0.329	0.585	0.445	0.777	0.408	1.482
Self-employed	−0.342	0.327	1.094	0.296	0.710	0.374	1.349
Unemployed	0.253	0.590	0.183	0.668	1.287	0.405	4.091
Mother’s occupation (Ref: Public sector workers)							
Blue-collar worker	0.023	0.352	0.004	0.948	1.023	0.514	2.038
Self-employed	0.157	0.353	0.196	0.658	1.169	0.585	2.337
Unemployed	−0.288	0.422	0.468	0.494	0.750	0.328	1.713
Father’s level of education (Ref: Lower secondary or below)							
Upper secondary	0.013	0.179	0.005	0.944	1.013	0.713	1.439
Bachelor’s degree or higher	0.258	0.277	0.869	0.351	1.294	0.752	2.227
Mother’s level of education (Ref: Lower secondary or below)							
Upper secondary	−0.142	0.174	0.671	0.413	0.867	0.617	1.219
Bachelor’s degree or higher	−0.216	0.294	0.538	0.463	0.806	0.453	1.434
Only child (yes/no) (Ref: yes)							
No	0.115	0.150	0.591	0.442	1.122	0.836	1.506
Marital status of parents (Ref: Intact family)							
Divorced/separated	0.110	0.220	0.251	0.616	1.116	0.726	1.717
Widowed	0.832	0.598	1.932	0.165	2.297	0.711	7.423
Family type (Ref: Mid-range)							
Balanced	0.331	0.193	2.958	0.085	1.393	0.955	2.031
Extreme	−0.308	0.187	2.716	0.099	0.735	0.509	1.060
DAS	0.012	0.004	11.413	0.001^**^	1.012	1.005	1.019
Family Cohesion	−0.015	0.015	0.975	0.323	0.985	0.956	1.015
Family Adaptability	−0.010	0.017	0.371	0.543	0.990	0.958	1.023
Social Support	−0.023	0.007	9.917	0.002^**^	0.977	0.963	0.991
Resilience	−0.030	0.014	4.514	0.034^*^	0.970	0.944	0.998
CBF-PI-B-Openness	0.014	0.013	1.217	0.270	1.014	0.989	1.041
CBF-PI-B-Conscientiousness	−0.003	0.014	0.045	0.832	0.997	0.969	1.026
CBF-PI-B-Extraversion	−0.004	0.013	0.078	0.780	0.996	0.971	1.023
CBF-PI-B-Agreeableness	−0.018	0.015	1.416	0.234	0.983	0.954	1.011
CBF-PI-B-Neuroticism	0.152	0.03	127.851	<0.001^***^	1.164	1.134	1.195
Constant	−1.585	1.081	2.148	0.143	0.205		

* *P* < 0.05; ** *P* < 0.01; *** *P* < 0.001.

## 4. Discussion

### 4.1. The current status of subthreshold depression among vocational nursing student in eastern China

A growing body of evidence underscores subthreshold depression as a prodromal manifestation of major depression, representing a widespread concern for global mental health. Using a CES-D cut-off score of ≥16, the prevalence of subthreshold depression in this study was 54.3%, showing both similarities and differences compared to related research. In 2018, a survey of 600 Jordanian university students by Dalky [39] found that 54.4% of participants reported mental health concerns, including various subthreshold depressive symptoms such as anxiety and stress. Similar trends have been observed in other regions. For instance, surveys conducted in Malaysia also indicated rising levels of depression, anxiety, and stress among college students. Furthermore, a study involving 2691 American college students revealed that more than one third experienced some degree of anxiety or depression during the COVID-19 pandemic [40].

Previous studies have documented the prevalence of subthreshold depression among adults and students at comprehensive universities/colleges in China, while research involving vocational school students remains scarce. According to Liao [41], the prevalence of subthreshold depression among adults in primary healthcare settings ranges from 2.9% to 9.9%, whereas community-based studies have reported rates between 1.4% and 17.2%. A subsequent study by Liao [42] conducted in Guangdong Province, China, identified an adult prevalence rate of 14.7%, lower than that observed among vocational students in this investigation. In this study, the prevalence of subthreshold depression among nursing students in vocational education was found to be relatively high. The variations observed in the detection rates of subthreshold depression may be partly attributable to differences in diagnostic criteria. For instance, while some studies use a CES-D score of 20 as the lower threshold for defining subthreshold depression, this study, consistent with the majority of the existing literature, adopts a cut-off score of 16 [14]. On the other hand, the differences may also be related to the characteristics of the sampled population. Vocational school students are typically in a transitional phase from adolescence to early adulthood. Research indicates that the prevalence of subthreshold depression increases significantly after the age of 12 and peaks during adolescence, between ages 14 and 16 [43]. Students in this group are in the late adolescent stage, during which both physical and psychological development are progressing from immaturity to maturity, while the nervous system remains relatively unstable. Furthermore, nursing students face a heavy academic burden, and the unique nature of their discipline exposes them to complex nurse–patient relationships, medical disputes, occupational injuries, and other challenges. Academic pressure combined with unknown associated factors may lead to an increase in negative emotions. When external stressors exceed their psychological coping capacity, they may be prone to extreme reactions. Within an exam-orientated education system, most schools neglect mental health education. As a result, students often lack effective strategies for managing intense emotions, which can lead to a range of mental health issues. Furthermore, compared to junior high or general high school students, vocational students are usually required to live on campus, away from their parents. The collective living environment can be difficult for many to adapt to, and conflicts may arise due to differences in lifestyle habits, leading to interpersonal difficulties and an increased susceptibility to depressive tendencies.

### 4.2. Differences between vocational nursing students with and without subthreshold depression

#### 4.2.1. Comparison of general demographic characteristics.

A comparison was made between individuals with subthreshold depression and those without, using chi-squared tests and t-tests to determine significant differences. The findings of the chi-squared test demonstrated significant disparities between the two groups with regard to gender, maternal level of educational attainment, parental marital status, and family type. The incidence of subthreshold depression was found to be significantly higher among female students than among their male counterparts. This finding is consistent with the results of previous studies [44,45]. The observed disparity may be attributed to women having greater susceptibility to emotionally triggering external factors, as well as the broader societal expectations placed on them, which contribute to increased academic and employment pressures. However, given the preponderance of women within the nursing profession, the possibility of sampling bias cannot be discounted. Further studies are required to validate these findings.

#### 4.2.2. Comparison of family functioning-related indicators.

This study found that students whose mothers had attained a higher level of education were less likely to experience subthreshold depression. However, no significant association was observed between fathers’ educational level and subthreshold depression. This pattern may be closely related to mothers’ familial roles, parenting approaches, and mental health awareness. Within the context of traditional Chinese social culture, the responsibility for child-rearing is shouldered by mothers in many households. Individuals with higher levels of education have been shown to have greater awareness of mental health issues, which may result in enhanced ability to recognise emotional distress in their children and provide timely and appropriate support. However, this does not imply that the role of the father is unimportant. A father’s level of education can indirectly affect his children via their mother. Consequently, it is necessary for both parents to pay close attention to their children’s mental health status and to assist in reducing negative emotions. Contrary to the findings of previous studies, our research demonstrated a higher prevalence of subthreshold depression among students from families characterised by harmonious parental marital relationships. This phenomenon may be attributed to the fact that families with stable marriages often have higher expectations for their children. Consequently, children who are influenced by this type of family atmosphere tend to pursue perfection, thereby experiencing greater academic or behavioural pressure. Additionally, children from families with harmonious marriages may have less exposure to adversity, rendering them more vulnerable to depressive emotions when facing difficulties [46]. Therefore, it is essential for parents to be attentive to their children#39;s emotional needs and to assist them in coping with stress. Based on our study, we hypothesise that family type exerts a certain influence on subthreshold depression (SD) among vocational nursing students. The SD group was primarily characterised by mid-range family types (44.5%). As demonstrated in Table 2, the levels of family cohesion and adaptability exhibited by students in the SD group were found to be significantly lower in comparison to those observed in the normal group. In accordance with the principles outlined by Olson’s [21] Circumplex Model of Marital and Family Systems, mid-range family typologies are distinguished by an imbalance between cohesion and adaptability. This implies that one dimension remains within the standard range, while the other exhibits an excessively high or low value, thereby signifying impairments in specific domains of family functionality. Subsequent analysis indicated that the three most prevalent family subtypes among nursing students with SD were Rigid-Separated (16.6%), Flexible-Enmeshed (15.3%), and Rigid-Disengaged (13.7%). Among these, extreme family types demonstrated the poorest overall functioning. The findings of this study indicate that within the SD group, family relationships are characterised by emotional independence, accompanied by the maintenance of appropriate boundaries. However, certain students demonstrated an excessive detachment from, or over-dependence on, their parents, reflecting either weak or highly entangled emotional connections. The transition of late adolescents towards independence is accompanied by a psychological shift that marks a departure from the authority of their parents. This process is frequently referred to as psychological ‘weaning’. This dynamic can result in reduced communication with parents, resistance against parental authority, and, in the most severe cases, emotional distancing. These developmental shifts frequently result in a reduction in familial cohesion. Conversely, some families exhibit a lack of clearly delineated personal boundaries, resulting in elevated levels of emotional and behavioural enmeshment, over-reliance on one another, and difficulties in independent decision-making or problem-solving.

Furthermore, families in the SD group demonstrated significantly lower levels of cohesion and adaptability, which resulted in impaired communication, emotional alienation, inflexible family roles, and an inability to adjust effectively to developmental changes or external stressors. These factors collectively compromise overall family functioning. In comparison to their counterparts enrolled at universities, vocational students generally encounter reduced academic pressure and more leisure time. However, it is important to note that many individuals become excessively engaged in online games, which has the potential to place further strain on family relationships. Some studies have indicated that internet addiction has the potential to reduce parent–child communication and shared daily experiences, which can result in family conflict resolution becoming more challenging [47]. This behaviour has been linked to delayed psychosocial maturation due to prolonged exposure to virtual environments. This may also contribute to the development of subthreshold depression in this population. The enhancement of family cohesion and adaptability has been demonstrated to be a means of reducing depressive symptoms and promoting physical health among adolescent students [35,36].

#### 4.2.3. Comparison of DAS, social support, resilience, and personality traits between the two groups.

As demonstrated in Table 2, the DAS score of nursing students in the SD group was found to be significantly higher than that in the non-SD group. Furthermore, nursing students who exhibited elevated levels of depressive symptoms also demonstrated a higher level of dysfunctional attitudes, a finding that is consistent with the results of previous studies. Dysfunctional attitudes have been observed to manifest with notable stability within the cognitive structure of adolescents. In the event of adverse life experiences, such as those previously outlined, the potential for the onset of dysfunctional attitudes in adolescents is increased. This can result in a tendency to interpret external information in a negative and distorted manner. This, in turn, gives rise to a series of depressive symptoms or emotions.

Additionally, the levels of social support and resilience were significantly lower in the SD group than in the control group. Numerous studies have demonstrated that a low level of social support is closely associated with an increased risk of depression. In circumstances where social support is inadequate, individuals may experience feelings of isolation and helplessness and find themselves lacking the necessary resources to cope with stress and adversity. This, in turn, can increase their vulnerability to depressive symptoms. Resilience has been identified as a critical protective factor for individuals’ mental health, as it has been demonstrated to serve as a buffer against the adverse effects of challenging circumstances. In line with the findings of earlier research, this study demonstrates a negative correlation between resilience and emotional disorders. Vocational nursing students constitute a relatively distinctive student group. Faced with demanding academic workloads, relatively low levels of academic achievement, high employment pressure, and the imminent responsibility of engaging in patient care and life-saving tasks, these students often experience a sustained high-pressure environment. Consequently, they demonstrate a heightened vulnerability to depressive symptomatology in comparison to the general population.

Regarding personality traits, the SD group demonstrated lower scores in comparison to the non-SD group across all factors, with the exception of the neuroticism dimension of the CBF-PI-B. Individuals who score higher for the neuroticism factor tend to be more susceptible to negative emotions such as anxiety and depression.

#### 4.2.4. Related factors in subthreshold depression among vocational nursing students in eastern China.

Multivariable binary logistic regression was employed in this study to analyse the associations between respondents’ demographic characteristics, scale scores, and the associated factors of subthreshold depression. Independent associations were further quantified using odds ratios (*OR*s) and 95% confidence intervals (*CI*s). Notably, all 95% *CI*s for these *OR*s excluded the null value of 1, reinforcing the statistical stability and reliability of these independent effects. Regression analysis revealed that subthreshold depression in nursing students in vocational schools is closely associated with gender, dysfunctional attitudes, CBF-PI-B-Neuroticism trait, social support and resilience. Compared with males, females have a significantly higher odds of depression. This phenomenon can be partly attributed to women’s tendency to exhibit heightened emotional intensity and sensitivity [48]. In line with this gender-related pattern, research has shown that female vocational nursing students are more likely to experience depression than their male counterparts. This phenomenon has been attributed to various factors, such as academic performance, physical appearance, lifestyle habits, and cumulative pressure from academic and daily life [49]. Further studies have indicated that this gender discrepancy also applies to subthreshold depression, with female students demonstrating a higher tendency toward this condition than their male counterparts. Therefore, teachers and parents should pay more attention to female students. Psychological counselling and other approaches can alleviate psychological pressure, provide timely assistance in resolving difficulties, and identify subthreshold depressive tendencies early on. Effective intervention should be implemented promptly for students exhibiting subthreshold depression. Dysfunctional attitudes were independently and significantly positive associated with SD. According to Beck’s cognitive model of depression, depressive symptoms can be promoted by dysfunctional attitudes when triggered by stressful life events [50]. There is ample evidence that the content of dysfunctional thinking is associated with vulnerability to major depression [51,52]. This study confirmed that, as a high-risk population for MDD, individuals with subthreshold depression exhibited a significant positive correlation between dysfunctional attitudes and subthreshold depression. Therefore, greater attention should be paid to the population with psychological subhealth, especially adolescents in the critical period of cognitive development. Structured cognitive interventions should be conducted among this group to improve their cognitive regulation ability, thereby preventing the pathological transition from subthreshold depression to MDD. Among the various personality trait dimensions, the CBF-PI-B-Neuroticism factor significantly influences the occurrence of subthreshold depression in nursing students. Those with high neuroticism scores experience negative emotions such as anxiety and depression more readily. They also exhibit heightened sensitivity to stress and emotional stimuli, display greater emotional volatility, and are more prone to negative emotional states. Concurrently, research indicates that neuroticism may be associated with functional abnormalities in certain neurotransmitters (such as serotonin), which are closely linked to the development of depression.

Furthermore, regression analysis showed that after controlling for potential confounding variables, social support and resilience both entered the regression equation and were independently and negatively associated with subthreshold depression. These findings extend the correlational results obtained in the previous comparative analysis and confirm that social support and resilience may serve as potential protective factors against subthreshold depressive symptoms, rather than merely reflecting group differences. The independent predictive roles of these two factors suggest that interventions targeting the enhancement of social support resources and the improvement of psychological resilience may effectively reduce the risk of subthreshold depression among vocational nursing students. Taken together, these results highlight the importance of integrating both external supportive resources and internal psychological capacities into preventive strategies for subthreshold depression in this high-risk population.

The factors influencing subthreshold depression among vocational college nursing students are multifaceted and complex. While earlier studies suggested that factors such as place of birth, academic year, and being an only child may impact subthreshold depression, this study found these to be non-significant. The regression equation revealed that gender and neuroticism were the only significant factors. This discrepancy may be attributed to the geographical location of the survey population and the particular educational levels involved. Further validation of the potential for sampling bias is required.

## 5. Conclusions

The main findings of this study can be summarised as follows. Firstly, the prevalence rate of subthreshold depression among nursing students in this study was found to be 54.3%, indicating a higher incidence compared to other groups. Secondly, factors associated with the subthreshold depression among Chinese vocational nursing students were identified, including demographic characteristics, basic family information, level of family functioning, dysfunctional attitudes, social support, resilience, and personality traits. Students exhibiting subthreshold depression demonstrate relatively unhealthy cognitive attitudes and heightened emotional sensitivity and instability, as well as lower levels of family functioning, social support, and psychological resilience. The results show that being female, scoring highly for dysfunctional attitudes and CBF-PI-B-Neuroticism, insufficient social support, and low psychological resilience are associated factors for the prevalence of subthreshold depression among Chinese vocational nursing students. This study suggests that various entities, including the state, community, hospitals, college, and family, should create and share resources and develop programmes to improve the mental health of Chinese vocational nursing students.
